# HPLC Analysis and Cytotoxicity of n-Butanol Extract from Glyphaea brevis Roots Against C6 Glioma Cells

**DOI:** 10.3797/scipharm.1307-08

**Published:** 2013-11-19

**Authors:** Koffi Marcel Konan, Janat Akhanovna Mamyrbekova-Bekro, Norbert Bakalara, David Virieux, Jean-Luc Pirat, Yves-Alain Bekro

**Affiliations:** 1 Laboratoire de Chimie Bioorganique et de Substances Naturelles (LCBOSN), UFR-SFA, Université Nangui Abrogoua, 02 BP 801 Abidjan 02, Côte d’Ivoire.; 2 UMR 5253, ICG Montpellier, Equipe AM2N, ENSCM, 8, rue de l’Ecole Normale, 34296 Montpellier cedex 5, France.; 3 INSERM U-1051, Institut des Neurosciences de Montpellier, 80 rue Augustin Fliche 34091 Montpellier, France.

**Keywords:** *Glyphaea brevis*, HPLC, Protocatechuic acid, Cytotoxicity, C6 Cells

## Abstract

The *n*-butanol extract of the roots of *Glyphaea brevis* was analysed. HPLC analysis suggested the presence of phenolic compounds like protocatechuic acid (PCA). The extract showed moderate cytotoxic activity against C6 glioma cells (EC_50_ > 1 mg/ml).

## Introduction

*Glyphaea brevis* (Spreng) Monachino (Malvaceae) is a spreading shrub, climber, or small tree up to 8 m high. It is very common in the undergrowth of closed forests, secondary jungles, on river-banks, lowlands, and in sub-mountain ecosystems, and is widespread in tropical Africa [1] where it is valued as a vegetable [2]. Various therapeutic uses such as the treatment of hepatitis, dyspepsia, ulcers, and poisoning have been reported. The root decoction is used as an aphrodisiac, appetizer, laxative, and as a remedy for chest pains, diarrhea, dysentery, and sleeping sickness. A beverage made from the root extracts obtained using water decoction, and associated with guinea grain consumption, is used to treat afflictions like paralysis [3, 4]. *G. brevis* showed considerable antibacterial, anti-inflammatory, and antioxidant activities [5, 6].

In this study, we evaluated the activity of *n*-butanol extracts of *G. brevis* roots on glioma cell proliferation as a contribution to the search for new anti-cancer strategies.

## Experimental

### Preparation of Plant Extracts

The plant material was collected on the site of the University of Abobo-Adjame (Abidjan, Côte d’Ivoire). It was previously identified in accordance with the available herbarium at the National Center Floristic (CNF) based in the University of Cocody with the help of botanists from this center.

Two hundred grams (200 g) of fine powder of the dried roots were mixed in 1 l of 70% (v/v) aqueous methanol under permanent agitation for 24 h at ambient temperature. The extract was filtered first through cotton wool. The extract was concentrated using a rotary evaporator in a water bath set at 65°C. The remaining concentrated extract (110 ml) was kept for 48 h in a refrigerator, then decanted, and was filtered again through filter paper. The remaining concentrated extract was extracted using three organic solvent extractions successively: 500 ml of hexane, 500 ml of chloroform, 500 ml of ethyl acetate, and 500 ml of *n*-butanol. The *n*-butanol fraction was used for this study.

### HPLC Analysis

The analytical HPLC used was equipped with an UV/vis detector set at 280 nm, a dC18 (5 μm) Atlantis 250 × 2 mm column at 25°C, and a binary gradient composed of solvent A (HCO_2_H/H_2_O, 2/98, v/v) and solvent B (CH_3_CN/HCO_2_H/H_2_O 80/2/18, v/v/v). The column flow rate was set at 0.25 ml/min^−1^ with a gradient program as shown in Table 1.

### Cell Cultures

The C6 glioma cell lines, originally cloned from an N-nitosomethylurea-induced glioma [7] were purchased from the American Type Culture Collection (Bethesda, MD, USA) and gifted by Dr G. Rebel (CNRS, Strasbourg). They were maintained in culture flasks with 5 ml of the culture medium containing 70% minimum essential medium (MEM) and 30% Hanks solution containing 5% fetal calf serum. The cells were incubated at 37°C under an atmosphere of air/CO_2_ (95/5). For the experiments, a pricking out was performed. After removal from the culture medium, cells were treated for 4 to 5 min with trypsin (0.1%) (Sigma–Aldrich). The cell suspension was then placed in 5 ml of fresh culture medium as stated above, and 50 μl was collected to perform the cell count. After dilution, the culture plates were seeded in 96-well plates; with the culture volume being 200 μl.

### MTT Viability Assay Test

The cells were treated with 100 μl of drug solution for concentrations ranging from 1 μg/ml to 0.5 μg/ml. Two well plates were treated for 24 h and 48 h. The operation was repeated for all of the desired concentrations.

The quantification of the live cells was performed according to the MTT (tetrazolium salt) test. After the expected incubation time, 15 μl of MTT solution (1 μg/ml) were added to each well and the mixture was incubated at 37°C for 2 h under a humid atmosphere balanced with 5% CO_2_. Each well was emptied and 100 μl of DMSO were added to the cell lysis; the intensity of the resulting color was directly related to the cell viability. This viability was quantified by measuring the absorbance at 570 nm using a plate reader 96-well, Bio-Rad Microplate Reader 550. Each 96-well plate contained a series of 12 wells not treated with the drug, used as a control. Thereafter, different reports of well absorbance/control absorbances were determined. This directly gave the percentage of live cells in the medium.

## Results and Discussion

Previously, the roots of *G. brevis* were extracted using hexane, chloroform, ethyl acetate, and n-butanol successively. The total phenolic content was determined by the Folin-Ciocalteu method. The extracts were tested for their antioxidant activity measuring the reduction of DPPH absorption to indicate the capacity to scavenge free radicals. The thin-layer chromatography method was developed for the qualitative components. We noticed the presence of polyphenols and particularly flavonoids in the studied extracts [5].

**Fig. 1:**
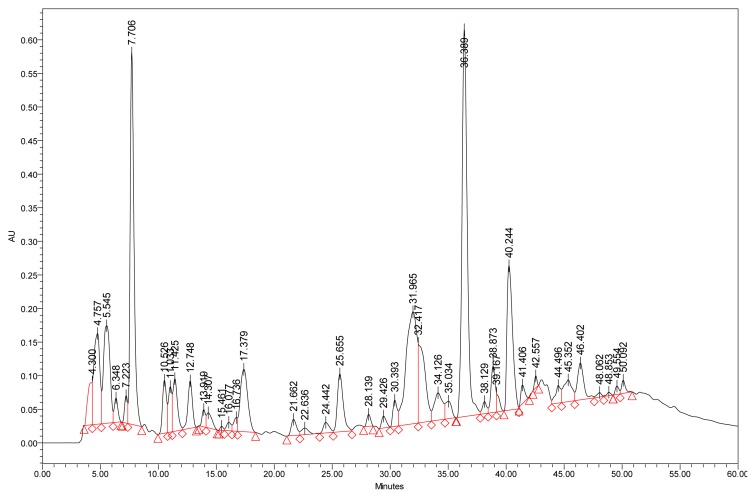
Chromatogram at 280 nm of n-butanol extract of *G. brevis* roots

In this study, we analyzed the *n*-butanol extracts of the roots of *G. brevis* by HPLC at 280 nm (Figure 1).

**Fig. 2:**
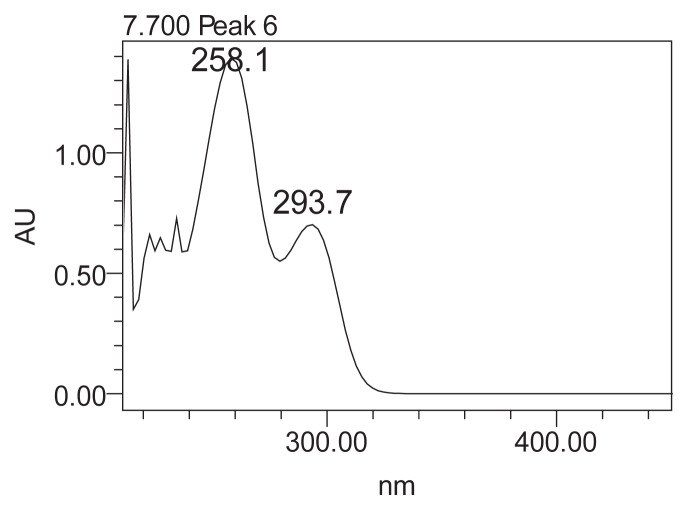
UV/vis spectrum of the compound at retention time of 7.7 min at 280 nm

The UV/vis spectra of each HPLC peak of Figure 1 were established. The peak observed in the studied sample at a retention time of 7.7 min at 280 nm shows two maxima (258 and 293 nm) which are characteristic of protocatechuic acid (Fig. 2).

**Fig. 3:**
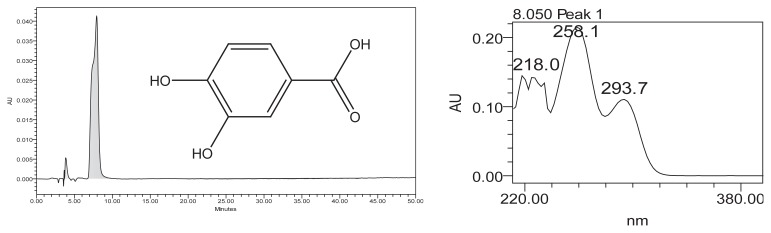
HPLC and UV/vis spectra of protocatechuic acid (PCA) at 280 nm

The max absorptions of the UV/vis spectra of the compound corresponding to the HPLC peak at RT = 7.7 min matched correctly with the maxima absorptions found in the UV/vis spectra of the protocatechuic acid standard solution (Fig. 3). The peak observed in the study sample is therefore protocatechuic acid with high probability.

**Fig. 4:**
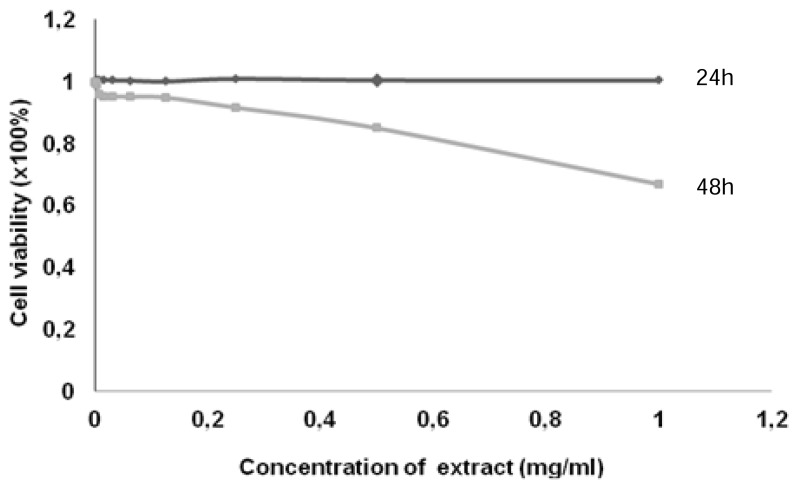
Cytotoxicity of *n*-butanol extract against C6 cells

The cytotoxicity study was carried out at a density of 3000 cells per well. The tests were performed after 24 and 48 hours of incubation. The percentages of inhibition of cell growth are presented in Figure 4.

After 24 h, the antiproliferative activity was not noticeable; the roots of *G. brevis* exhibited low activity against the C6 glioma cells. After 48 h, we observed a depletion of the cell line. There was a gradual decrease in the cell population; a dose-response relationship was observed.

In our case, the percentage of inhibition of C6 cells did not reach 50% even with a maximum of 1 mg/ml. Therefore, the *n*-butanol extract of the roots of *G. brevis* is not endowed with a good antiproliferative activity. However, this fraction contains cytotoxic compounds with respect to the dose-response activity observed after 48 h of incubation. This observed effect may be due to the presence of the plant’s secondary metabolites including protocatechuic acid. Protocatechuic acid has been reported to induce apoptosis of human leukemia cells [8].

## Conclusion

The present study highlights the cytotoxic potential of *G. brevis*. The dose-response effect of n-butanol extract of the roots of *G. brevis* indicates that this plant contains potentially active compounds. The isolation of different compounds and the study of structure-activity will identify the cytotoxic potency of the roots of this plant. Given the wide range that cancer covers, it would be interesting to evaluate the antiproliferative activity of *G. brevis* on the viability of other cancer cells.

## Figures and Tables

**Tab. 1:** Gradient mode during 75 min

Time (min)	0	2	5	12	15	25	40	45	55	70	75
%A	100	100	98	98	97	92	80	75	75	35	35
